# Metformin Collaborates with PINK1/Mfn2 Overexpression to Prevent Cardiac Injury by Improving Mitochondrial Function

**DOI:** 10.3390/biology12040582

**Published:** 2023-04-11

**Authors:** Zhuang Ma, Zuheng Liu, Xudong Li, Hao Zhang, Dunzheng Han, Wenjun Xiong, Haobin Zhou, Xi Yang, Qingchun Zeng, Hao Ren, Dingli Xu

**Affiliations:** 1 State Key Laboratory of Organ Failure Research, Department of Cardiology, Nanfang Hospital, Southern Medical University, Guangzhou 510080, China; 2 Key Laboratory for Organ Failure Research, Ministry of Education of the People’s Republic of China, Guangzhou 510515, China; 3 Xiamen Key Laboratory of Cardiac Electrophysiology, Department of Cardiology, Xiamen Institute of Cardiovascular Diseases, The First Affiliated Hospital of Xiamen University, School of Medicine, Xiamen University, Xiamen 361013, China; 4 Department of Cardiology, The First Affiliated Hospital of Guangzhou Medical University, Guangzhou 510120, China; 5 Department of Rheumatology, Nanfang Hospital, Southern Medical University, Guangzhou 516006, China

**Keywords:** mitochondria, PINK1, Mfn2, PGC-1α, metformin, isoprenaline

## Abstract

**Simple Summary:**

Mitochondria are seriously fragmented, damaged and accompanied by insufficient productivity during heart failure. Studies have shown that PINK1 can mediate mitophagy to clear damaged mitochondria, activation of PGC-1a can promote mitochondrial regeneration, and Mfn2 can promote mitochondrial fusion. Therefore, in this study, we investigated the effect of PINK1 overexpression on myocardial mitophagy and the effect of comprehensive improvement of mitochondrial quality on injured cardiomyocytes. Our results show that overexpression of PINK1 can alleviate myocardial injury through mitophagy, while promoting mitochondrial regeneration and fusion can further improve cardiomyocyte function.

**Abstract:**

Both mitochondrial quality control and energy metabolism are critical in maintaining the physiological function of cardiomyocytes. When damaged mitochondria fail to be repaired, cardiomyocytes initiate a process referred to as mitophagy to clear defective mitochondria, and studies have shown that PTEN-induced putative kinase 1 (PINK1) plays an important role in this process. In addition, previous studies indicated that peroxisome proliferator-activated receptor gamma coactivator-1α (PGC-1α) is a transcriptional coactivator that promotes mitochondrial energy metabolism, and mitofusin 2 (Mfn2) promotes mitochondrial fusion, which is beneficial for cardiomyocytes. Thus, an integration strategy involving mitochondrial biogenesis and mitophagy might contribute to improved cardiomyocyte function. We studied the function of PINK1 in mitophagy in isoproterenol (Iso)-induced cardiomyocyte injury and transverse aortic constriction (TAC)-induced myocardial hypertrophy. Adenovirus vectors were used to induce PINK1/Mfn2 protein overexpression. Cardiomyocytes treated with isoproterenol (Iso) expressed high levels of PINK1 and low levels of Mfn2, and the changes were time dependent. PINK1 overexpression promoted mitophagy, attenuated the Iso-induced reduction in MMP, and reduced ROS production and the apoptotic rate. Cardiac-specific overexpression of PINK1 improved cardiac function, attenuated pressure overload-induced cardiac hypertrophy and fibrosis, and facilitated myocardial mitophagy in TAC mice. Moreover, metformin treatment and PINK1/Mfn2 overexpression reduced mitochondrial dysfunction by inhibiting ROS generation leading to an increase in both ATP production and mitochondrial membrane potential in Iso-induced cardiomyocyte injury. Our findings indicate that a combination strategy may help ameliorate myocardial injury by improving mitochondrial quality.

## 1. Introduction

Activation of the sympathetic nervous system is a vital adaptive response that increases myocardial contractility and heart rate, which can increase cardiac output during the early period of induced stress [1,2]. However, persistent stress can lead to myocardial hypertrophy, ultimately resulting in myocardial remodeling and heart failure [3,4]. Activation of the sympathetic nervous system is crucial because it triggers myocardial hypertrophy. Moreover, cardiac hypertrophy is accompanied by mitochondrial dysfunction [5]. Thus, maintaining the homeostasis of mitochondrial metabolism is essential for the normal physiology of a healthy heart. To prevent mitochondrial dysfunction, cardiomyocytes engage effective preventive mechanisms that maintain mitochondrial homeostasis by regulating mitochondrial biogenesis and mitochondrial autophagy [6,7,8]. In addition, since mitochondria are highly dynamic organelles that are ceaselessly remodeled, which results in changing size, shape and number to meet energy demands, more attention has been directed to improving overall mitochondrial quality control.

Peroxisome proliferator-activated receptor gamma coactivator-1α (PGC-1α) is a powerful transcriptional coactivator in energy metabolism [9]. Emerging evidence indicates that PGC-1α ameliorates cardiac injury by increasing fatty acid oxidation, mitochondrial regeneration and antioxidant responses to stress [10,11]. As a cotranscriptional regulatory factor, PGC-1α stimulates mitochondrial regeneration by inducing NRF-1 and NRF-2 gene expression, which promotes the expression of Tfam [12]. In addition, metformin protects cells by promoting mitochondrial regeneration via the PGC-1α signaling pathway [13]. Thus, metformin was selected to promote the biogenesis of new healthy mitochondria to replenish injured mitochondria and meet the requirement of biological energy.

PTEN-induced putative kinase 1 (PINK1) is a crucial protein involved in mitochondrial quality control because of its participation in the mitophagy process [14,15]. Previous studies have shown that PINK1 is downregulated in failing hearts. Moreover, PINK1 knockdown was closely linked to the occurrence of cardiac hypertrophy [16]. Our previous findings suggested that PINK1 might play a protective role in cardiomyocytes exposed to angiotensin II [17]. In addition, PINK1 phosphorylates mitofusin 2 (Mfn2) to recruit Parkin and initiates mitophagy, an essential mitochondrial homeostasis mechanism [18]. Mfn2, a transmembrane GTPase, is located at the outer membrane of mitochondria and the endoplasmic reticulum [19]. Knocking out Mfn2 expression increased the apoptosis rate of cells under both normal and hypoxic conditions, and Mfn2 overexpression reduced hypoxia-induced apoptosis [20,21,22]. In addition, the inhibition of mitochondrial fusion reduced myocardial contractility despite calcium and potassium stimulation [23,24]. More importantly, Mfn2 knockout led to a reduction in both mitochondrial respiration and ATP production, as well as reduced oxidative phosphorylation [25,26,27]. Notably, PGC-1α knockout led to a decrease in Mfn2 levels, whereas Mfn2 knockout inhibited PGC-1α expression [28,29]. These findings suggest a potential role for Mfn2 in the metabolism and biogenesis of mitochondria.

In this study, we speculated that a combined strategy of mitochondrial biogenesis with metformin treatment and mitophagy induction mediated by the PINK1/Mfn2 pathway might be beneficial for ameliorating cardiomyocyte injury.

## 2. Materials and Methods

### 2.1. Cell Culture and Adenoviral Transduction

All animal experiments were administrated in accordance with the guidelines of the Declaration of Helsinki and were approved by the Institutional Review Board of Southern Medical University (approval number: L2019053). Specific-pathogen-free (SPF) one-day-old Sprague Dawley neonatal rats were purchased from Southern Medical University Laboratory Animal Technology Development Co., Ltd. All animal experiments were conducted in the experimental animal room of the Southern Medical University Health Science Center. One-day-old neonatal rats were euthanized by 2% isoflurane inhalation and cervical dislocation. The heart was then excised and digested with 0.25% trypsin (Gibco) at 4 °C for 12–16 h, and then digested with 3–5 mL type II collagenase (Gibco) at 36 °C, 200 RPM for 15 min. The cell suspension was collected and centrifuged (1000 rpm, 5 min). Subsequently, the cell suspension was inoculated into cell culture dishes and placed in a cell incubator at 5% CO_2_ and 37 °C for about 1.5 h. The supernatant was then collected and seeded in suitable petri dishes. Neonatal rat ventricular myocytes (NRVMs) were cultured as previously described [30]. Adenoviral vectors harboring PINK1 (Ad-Pink1) were synthesized by GeneChem Co. (Shanghai, China), and adenovirus vectors harboring Mfn2 (Ad-Mfn2) were synthesized by Obio Technology Company (Shanghai, China). The multiplicity of infection (MOI) ranged from 1 to 10. The viruses were added to cells per the respective manufacturer’s instructions. In brief, NRVMs were extracted and inoculated with 1 × 10^5^~5 × 10^5^ cells into 12-well plates containing appropriate medium for 48 h. Most of the cardiomyocytes were beating in sheets and in good shape. At this time, the degree of cell fusion was about 80%. According to the setting of virus transfection complex gradient, the corresponding amount of adenovirus was dissolved in serum-free DMEM medium, and then the same volume of serum-containing DMEM medium was added 2 h later. After adenoviral transduction for 72 h, NRVMs were stimulated with 10 μM Iso for 48 h, and then subsequent experiments were performed.

### 2.2. Generation of Mice with Cardiac-Specific Overexpression of PINK1

The Cre-dependent Cas9 knock-in mouse model was purchased from Model Organisms (Shanghai, China). The CRISPR/Cas9 technique was engaged in inserting the CAG-LSL-Pink1-3XFlag-Wpre-pA expression box at the Rosa26 gene locus by homologous recombination. A schematic diagram of the mouse construction strategy is shown in Figure S1A in the Supplementary Materials, and the brief process is as follows: Cas9 mRNA and gRNA were obtained by in vitro transcription; a donor vector was established through In-Fusion cloning technology. The donor vector comprised a 3.3 kb 5′ homologous arm, Cac-Lsl-Pink1-3xflag-wpre-Pa, and a 3.3 kb 3′ homologous arm. gRNA, Cas9 mRNA and donor vector were microinjected into the fertilized eggs of C57BL/6J mice to obtain F0 generation mice. The obtained mice were then crossed with α-MHC-Cre mice. Mice were injected with tamoxifen for 5 days to achieve cardiac-specific PINK1 overexpression. A typical graph of DNA by running gel electrophoresis is shown in Figure S1B in the Supplementary Materials.

### 2.3. Echocardiography

We performed echocardiography 8 weeks after surgery. In brief, mice were anesthetized with isofluoride gas, fixed on an ultrasonic workbench in the supine position, coated with conductive fluid on limbs, hair removal cream was used to remove the hair in the precardiac area, the electrocardiogram was connected, and then an appropriate amount of ultrasonic coupling agent was applied. The isoflurane dose was adjusted to keep the mice under light anesthesia and the heart rate was controlled at 400–500 beats per minute. Note that either too slow or too fast a heart rate can affect the ultrasound results. Mouse heart function and structure were evaluated using a 30 Hz high-frequency probe using small animal ultrasound (Vevo 2100, Fujifilm Visual Sonics, Toronto, ON, Canada). The images and data of the long and short axis of the mouse heart were obtained by adjusting the angle and position of the ultrasonic probe.

### 2.4. Chemicals and Reagents

Dulbecco’s modified Eagle’s medium (DMEM) and fetal bovine serum (FBS) were purchased from Gibco (Big Cabin, OK, USA). Collagenase and trypsin were purchased from Sigma (St. Louis, MO, USA). Iso and metformin were purchased from Sigma. The following antibodies were used in this study: anti-Mfn2, anti-PINK1 (Abcam, Cambridge, UK), anti-Beclin1, anti-P62, anti-LC3B, anti-PGC 1α, anti-TFAM, anti-NRF1, anti-ANP, anti-β-MHC, anti-Col-1, anti-TGF-β-1, anti-GAPDH and anti-β-actin (Proteintech, Chicago, IL, USA).

### 2.5. Transmission Electron Microscopy (TEM)

The medium containing the serum was removed by aspiration, and 1 mL serum-free medium or PBS was added to scrape off the cells. Then, the cells were transferred into a 1.5 mL apical bottom Ep tube and centrifuged at 1000 rpm for 5 min. A 2.5% glutaraldehyde working solution 300–500 µL was added into the Ep tube and fixed at room temperature for 1 h. After fixation at 4 °C for 3 h or overnight, replace glutaraldehyde with PBS and fill Ep tube. Subsequently, the cells were postfixed in 1% osmium tetroxide for 90 min, dehydrated in a graded series of ethanol concentrations, and embedded in Spar resin. Ultrathin sections (50–70 nm) were sliced with an ultramicrotome (Leica, Weztlar, Germany) and contrast stained with uranyl acetate and lead citrate. Samples were visualized with an electron microscope (JEM-1400, Tokyo, Japan).

### 2.6. Evaluation of the Colocalization of Mitochondria and Lysosomes

The colocalization of lysosomes and mitochondria was visualized to examine mitophagy. Cardiomyocytes were coincubated with MitoTracker Green (100 nM) and LysoTracker Red (50 µM, Molecular Probes, Eugene, OR, USA) at room temperature for half an hour. Subsequently, nuclei were stained with Hoechst for 10 min. The colocalization was examined with a confocal microscope (Leica, Germany). Bright red fluorescence was used to highlight lysosomes, and blue fluorescence represented cell nuclei. Bright orange fluorescence indicates the area where mitochondria and lysosomes overlap, which indirectly represents mitophagy.

### 2.7. ROS Determination

Cellular ROS production was measured with 2,7-dichlorodihydrofluorescein diacetate (DCFH, Beyotime, Shanghai, China), purchased from Beyotime, following the manufacturer’s protocol. Cardiomyocytes were cultured in a confocal dish and incubated in culture medium containing 10 μM DCFH-DA for half an hour at room temperature. A fluorescence microscope (Leica, Germany) was used to detect ROS production. Bright green fluorescence represents ROS. Image-Pro Plus was used for the analysis.

### 2.8. TUNEL Assay

A TdT-mediated dUTP nick-end labeling (TUNEL) assay (Beyotime, China) was used to examine the apoptosis rate of cardiomyocytes per the manufacturer’s protocol. Cardiomyocytes were fixed with 4% paraformaldehyde for 30 min, and then 0.1% Triton X-100 PBS was added for 10 min at room temperature. Then, cardiomyocytes were incubated with TUNEL solution at room temperature for 1 h. Apoptotic morphological features were imaged using a fluorescence microscope (Leica, Germany). Image-Pro Plus was used for the analysis.

### 2.9. MMP Determination

The mitochondrial membrane potential (MMP) was measured using a JC-1 Mitochondrial Membrane Potential Assay Kit, purchased from Beyotime, following the manufacturer’s protocol. Briefly, cardiomyocytes were incubated with JC-1 staining solution in the dark for 20 min at 37 °C. The fluorescent images were imaged using a fluorescence microscope (Leica, Germany) by red and green fluorescence. Image-Pro Plus was used for analysis.

### 2.10. ATP Assay

The ATP contents of cardiomyocytes were evaluated using a Firefly Luciferase ATP Assay Kit, purchased from Beyotime, following the manufacturer’s protocol. Briefly, the culture medium was aspirated, and the cells were lysed by adding 200 µL of lysate to each well of a 6-well plate. To fully lyse the cells, a pipette was used to repeatedly blow or shake the culture plate to fully contact the lysate and lyse the cells. After lysis, the lysate was centrifuged at 12,000× *g* for 5 min at 4 °C, and the supernatant was retained for subsequent determination. The reagents to be used were melted in an ice bath, and ATP standard solutions were diluted with ATP assay lysates to concentrations of 0.01, 0.03, 0.1, 0.3, 1, 3 and 10 µM. Then, 100 µL of ATP test solution was added to the test well for 5 min at room temperature. Twenty microliters of sample or standard was added to test wells, mixed quickly, and after at least 2 s, RLU or CPM was measured with a luminometer. The level of ATP was detected using a Multi-Mode Detection Luminometer.

### 2.11. Cell Viability Assay

Cell viability was measured using a Cell Counting Kit-8 (CCK-8), purchased from Beyotime, following the manufacturer’s protocol. Briefly, neonatal cardiomyocytes were inoculated into 96-well plates at a density of 5000 cells per well. After stimulation, 20 µL of enhanced CCK-8 reagent was added to each well. Wells supplemented with corresponding amounts of cell culture medium and enhanced CCK-8 solution without the addition of cells were used as blank controls. The absorbance at 450 nm was detected with a microplate reader after 2 h of further incubation in the cell incubator.

### 2.12. Evaluation of Mitochondrial Respiration

Cardiomyocytes were seeded in a 96-well culture plate. Oxygen consumption was measured using extracellular oxygen consumption assay kits, purchased from Abcam, following the manufacturer’s protocol. Briefly, neonatal cardiomyocytes were inoculated in 96-well plates. After corresponding stimulation, 10 µL reconstituted Extracellular O_2_ Consumption Reagent or PBS was added to the replicate wells. Subsequently, 100 µL prewarmed high-sensitivity mineral oil was promptly added to all wells. The plate was immediately read in a fluorescence plate reader over 30 min (read every 2–3 min). The signal control well and blank control well readings (linear phase) were determined, and the signal-to-blank (S:B) ratio was calculated. The oxygen consumption rate was calculated to assess mitochondrial respiration.

### 2.13. Immunoblot Analysis

Cardiomyocytes were lysed with radioimmunoprecipitation assay (RIPA) lysis buffer (Beyotime, Shanghai, China) with phosphatase and protease inhibitors (Fdbio, Hangzhou, China). Then, the protein concentration was calculated by a BCA assay (Thermo Fisher, Waltham, MA, USA). The primary antibodies used included anti-PINK1 antibody and anti-Mfn2 antibody (1:1000, Abcam, Cambridge, MA, USA); anti-Beclin1 antibody, anti-P62 antibody, anti-LC3B antibody, anti-PGC 1α antibody, anti-TFAM antibody, anti-NRF1 antibody, anti-ANP antibody, anti-β-MHC antibody, anti-Col-1 antibody and anti-TGF-β-1 antibody (1:1000, Proteintech, Chicago, IL, USA); anti-β-actin antibody and anti-GAPDH antibody (1:5000, Proteintech, Chicago, IL, USA). The secondary antibody used was goat anti-rabbit IgG-HRP (1:5000, Proteintech, Chicago, IL, USA). The protein bands were examined with ECL substrate (Fdbio, Hangzhou, China), visualized by the Gene Gnome Imaging System (Syngene Bio Imaging, Cambridge, MA, USA) and quantified by densitometry with ImageJ software.

### 2.14. Statistical Analysis

Quantitative data are expressed as the means ± SDs. In order to test the normality of the data distribution, Shapiro–Wilk tests were performed. Statistical analyses were performed by one-way ANOVAs followed by Bonferroni or Dunnett’s test post-hoc tests. All statistical analyses were performed using SPSS Statistics 20.0. *p*-values < 0.05 were considered statistically significant.

## 3. Results

### 3.1. PINK1 Overexpression Increased Mitophagy in Iso-Treated Cardiomyocytes

**Figure 1:**
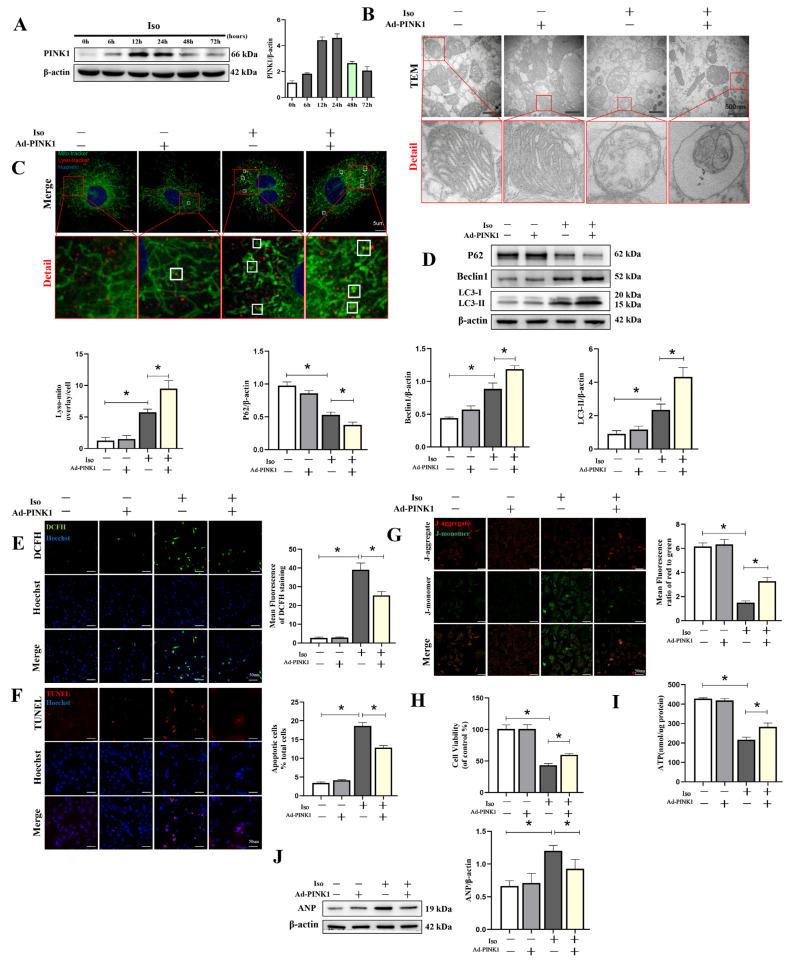
Overexpression of PINK1 attenuated isoprenaline-induced cardiomyocyte injury by mitophagy. Adenoviruses Ad-PINK1 and Ad-control were used to transfect cardiomyocytes and then stimulated with Iso (10 uM) for 48 h. (**A**) Western blot showing the expression of PINK1 over time. (**B**) Mitochondrial morphology was shown by TEM in cardiomyocytes. Details of mitochondrial structures are shown in the red frame. (**C**) Representative immunofluorescence images of lysosomal–mitochondrial interactions (yellow). Lysosomes are shown in red, mitochondria are shown in green, and nuclei are shown in blue. (**D**) Western blot showing the expression levels of P62, Beclin1 and LC3 II in each group. Quantification relative to β-actin levels. (**E**) DCFH staining was used to show ROS production (green) in cardiomyocytes. (**F**) Apoptotic cardiomyocytes (red) were examined by TUNEL staining. (**G**) Fluorescence images of MMP detected by JC-1 tracker. J-aggregate staining is shown in red and J-monomer staining is shown in green. (**H**) Cell viability was detected by the Cell Counting Kit-8 (CCK-8). (**I**) ATP assay kit with a luminometer was used to determine intracellular ATP levels. (**J**) Immunoblotting showing the expression of ANP in cardiomyocytes. Statistical analyses (**A**–**J**) were performed by one-way ANOVAs followed by Dunnett’s test post-hoc tests. Data are presented as the mean ± SD. (n = 3). * *p* < 0.05.

Heart failure is commonly accompanied by neuroendocrine system dysfunction. In addition, the effect of β1AA can be inhibited by atenolol, an antagonist of β1 adrenoceptors (β1AR), and imitated by isoprenaline, an agonist of β1AR. With increased Iso stimulation time, the expression of PINK1 peaked at 12 h, followed by stable expression until 24 h and decreased expression at 48 h. (Figure 1A, File S1). To evaluate the effects of PINK1 overexpression on mitophagy in an Iso-induced cardiomyocyte injury model, we detected mitochondrial changes by transmission electron microscopy (TEM) and confocal microscopy. TEM revealed that PINK1 overexpression increased the formation of autophagosomes (Figure 1B). Fluorescence images of the colocalized lysosomes and mitochondria visualized through microscopy (Figure 1C) indicated that PINK1 overexpression increased lysosomal–mitochondrial interactions (9.5 ± 1.29, *p* < 0.01) compared with the Iso-treated group (5.75 ± 0.5), which indicated enhanced mitophagy. This finding was supported by the results of Western blotting measuring autophagy-related proteins, including P62 (0.38 ± 0.04 vs. 0.53 ± 0.04, *p* < 0.05), Beclin1 (1.19 ± 0.05 vs. 0.89 ± 0.09, *p* < 0.05) and LC3 (4.32 ± 0.56 vs. 2.34 ± 0.35, *p* < 0.05) (Figure 1D, File S1). In addition, PINK1 overexpression reduced ROS generation (25.34 ± 2.08 vs. 39.0 ± 3.61, *p* < 0.01) and apoptosis (12.84 ± 0.59 vs. 18.64 ± 0.91, *p* < 0.01), as determined by DCFH and TUNEL staining (Figure 1E,F). These effects might have been due to the improvement of mitochondrial function in Iso-treated cells, as the mitochondrial membrane potential was reversed by PINK1 overexpression (3.28 ± 0.31 vs. 1.50 ± 0.16, *p* < 0.01) (Figure 1G). Additionally, the findings were supported by cell viability (59.9 ± 2.97 vs. 43.34 ± 2.65, *p* < 0.001) and ATP generation assays (282.75 ± 20.03 nmol/ug vs. 216.75 ± 13.53, *p* < 0.01) (Figure 1H,I), suggesting a reasonable explanation for the ANP, a cardiomyocyte hypertrophy marker protein, inhibition induced by PINK1 overexpression (Figure 1J, File S1).

### 3.2. Cardiac-Specific Overexpression of PINK1 Attenuated Pressure Overload-Induced Cardiac Hypertrophy and Fibrosis

**Figure 2:**
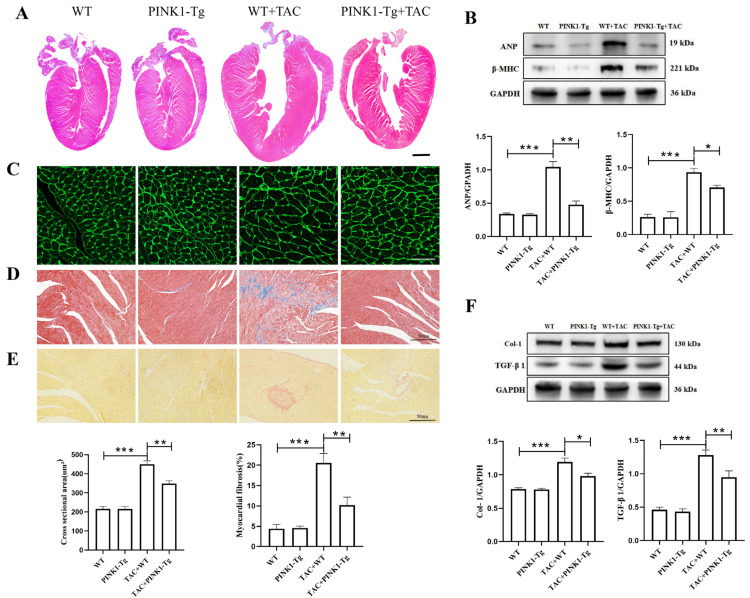
Cardiac-specific overexpression of PINK1 alleviated myocardial hypertrophy and fibrosis induced by transverse aortic constriction (TAC). Cardiac hypertrophy reflected by (**A**) HE staining in global and (**B**) protein expression of ANP and β-MHC (n = 3), and (**C**) WGA staining (n = 6). Collagen quantification was measured on Masson staining (**D**), Sirius Red staining (**E**) in detail (n = 6), and (**F**) protein (n = 3) expression of collagen 1 and TGF-β1; statistical analyses (**A**–**F**) were performed by one-way ANOVAs followed by Bonferroni test post-hoc tests. *: *p* < 0.05, **: *p* < 0.01, ***: *p* < 0.001. Data are presented as the mean ± SD.

Evidence has shown that PINK1 is involved in the mitophagy process, compresses morbidity and leads to health benefits in mice. Mice with cardiac-specific overexpression of PINK1 were constructed and subjected to TAC or sham operation. The peak flow rate ≥3.5 m/s at the aortic banding site was set as an indicator of successful aortic constriction (Figure S2 in the Supplementary Materials). Eight weeks after surgery, pathological cardiac hypertrophy in the TAC mice was mitigated by PINK1 overexpression (Figure 2A). Compared to the sham mice, the HW:BW ratio and HW:TL ratio in the TAC mice increased by 68.47% and 73.57%, respectively (Figure S3 in the Supplementary Materials). The protein levels of cardiac hypertrophy marker proteins ANP and β-MHC in the TAC mice were 3.06-fold and 3.58-fold higher, respectively, than those in the sham group (*p* < 0.001) (Figure 2B, File S1). WGA staining showed that compared to the WT mice, the average cross-sectional area of the cardiomyocytes was 109.4% larger in the TAC mice (*p* < 0.001) (Figure 2C). However, after overexpression of PINK1, the mean cardiomyocyte cross-sectional area decreased by 22.45% (*p* < 0.01). The HW:BW, HW:TL and the protein levels of ANP and β-MHC were significantly reduced. These results suggest that the overexpression of PINK1 attenuates TAC-induced myocardial hypertrophy. Cardiac fibrosis is associated with cardiac dysfunction and is usually an indication of structural remodeling. Masson (Figure 2D) and Sirius Red (Figure 2E) staining were used to detect the degree of myocardial fibrosis. Compared to the sham group, the fibrotic area was significantly increased in the TAC mice (*p* < 0.001), which was dramatically decreased by PINK1 overexpression. Previous research has shown that transforming growth factor-β1 (TGF-β1) plays a vital role in myocardial fibrogenesis. Immunoblotting showed that the expression levels of Collagen 1 and TGF-β1 were significantly increased in the TAC group (*p* < 0.001), but overexpression of PINK1 attenuated the expression of Collagen 1 and TGF-β1 (Figure 2F, File S1). These results suggest that PINK1 overexpression can inhibit cardiac fibrosis through affecting a TGF-β1-related signaling pathway induced by pressure overload.

### 3.3. PINK1 Overexpression Improved Cardiac Function and Facilitated Myocardial Mitophagy in TAC Mice

**Figure 3:**
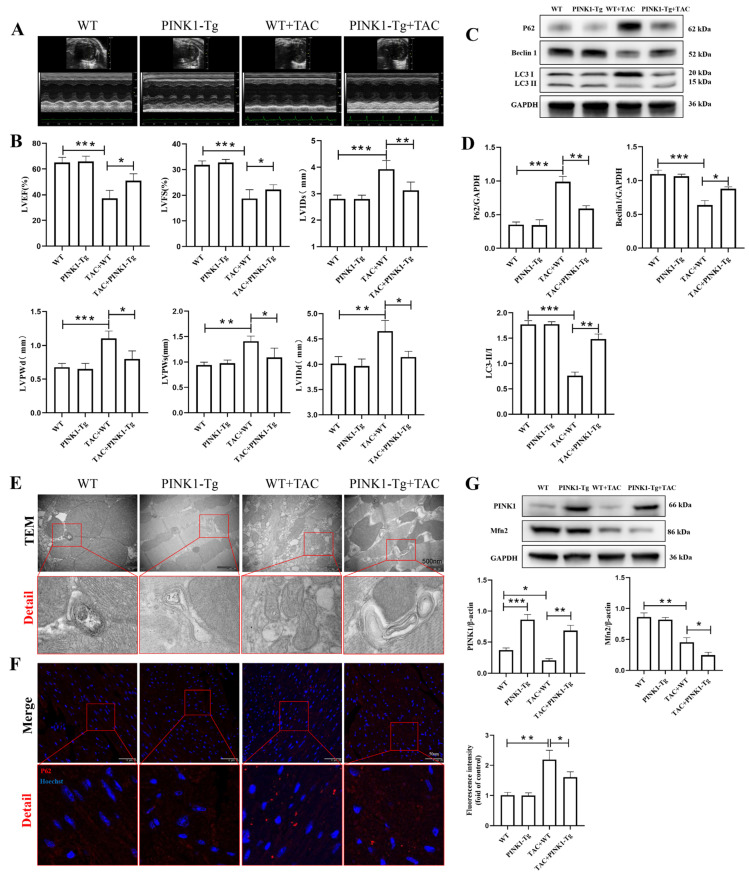
Effect of PINK1 overexpression on cardiac function and mitophagy in TAC mice. The echocardiography (ECG) was administrated at 8 w after surgery (**A**,**B**): LV ejection fraction (EF), LV fraction shortening (FS), left ventricular internal dimension in systole (LVIDs), left ventricular internal dimension in diastole (LVIDd), left ventricular posterior wall in systole (LVPWs), left ventricular posterior wall in diastole (LVPWd). n = 6 for each group. (**C**,**D**) Immunoblotting showing the expression of P62, Beclin 1 and LC3II in WT and PINK1-Tg mice. Densitometric analyses of the Western blotting results, P62 to GAPDH, Beclin1 to GAPDH, and LC3II to LC3I. (**E**) The autophagosome was shown by TEM in myocardial tissues. Details of mitochondrial structures are shown in the red frame (scale bars 500 nm). (**F**) Immunofluorescence staining of p62 expression (red) in myocardial tissues (scale bars 50 μm). (**G**) Immunoblotting showing the expression of PINK1 and Mfn2 in WT and PINK1-Tg mice. Statistical analyses (**A**–**G**) were performed using one-way ANOVAs followed by Bonferroni test post-hoc tests. *: *p* < 0.05, **: *p* < 0.01, ***: *p* < 0.001, values are presented as the mean ± SD, n = 3.

Previous studies have found that TAC can increase cardiac afterload and lead to cardiac remodeling and dysfunction. Therefore, we examined the effect of PINK1 on cardiac function. Echocardiography showed that eight weeks after TAC, LVEF and LVFS were significantly decreased compared to those in the sham mice (Figure 3A,B). The LVIDd, LVPWd, LVIDs, LVPWs and thicknesses were increased, indicating that pressure overload induced LV remodeling, cardiac hypertrophy and dysfunction. Meanwhile, overexpression of PINK1 in the myocardia increased LVEF and LVFS compared to the TAC mice. In addition, the PINK1-Tg + TAC group showed a smaller decrease in LVIDd (4.66 ± 0.21 mm vs. 4.14 ± 0.11, *p* < 0.001), LVIDs (3.93 ± 0.33 mm vs. 3.13 ± 0.31, *p* < 0.001), LVPWd (1.1 ± 0.11 mm vs. 0.8 ± 0.12, *p* < 0.001) and LVPWs (1.41 ± 0.11 mm vs. 1.09 ± 0.18, *p* < 0.01) than the WT + TAC group. These results strongly suggested that PINK1 overexpression protected TAC mice from pressure overload-induced cardiac dysfunction. Previous studies have shown that PINK1 is a vital protein involved in the mitophagy process, and its knockdown is closely related to the occurrence of cardiac hypertrophy. We detected the effects of PINK1 overexpression on mitophagy in TAC mice. The autophagy- and mitophagy-related Beclin1 and LC3 II protein levels in the TAC mice were 0.58-fold and 0.43-fold lower, respectively, and P62 protein levels were 2.83-fold higher than those in the sham group (*p* < 0.01) (Figure 3C,D, File S1). However, PINK1 overexpression improved the TAC-induced reduction in the expression of Beclin1 (0.64 ± 0.07 vs. 0.88 ± 0.03, *p* < 0.01) and LC3B II (0.76 ± 0.07 vs. 1.48 ± 0.1, *p* < 0.001) and reduced the expression of p62 (0.99 ± 0.08 vs. 0.59 ± 0.04, *p* < 0.001). In addition, transmission electron microscopy showed that the autophagosome was decreased after TAC but increased after PINK1 overexpression (Figure 3E). Furthermore, immunofluorescence staining revealed that hearts from TAC mice displayed more profound aggregation of p62 which decreased after PINK1 overexpression (Figure 3F). Use of CRISPR/Cas9 technique-mediated overexpression of the PINK1 protein induced cardiac overexpression of PINK1 (Figure 3G, File S1). As previously reported, we also found that myocardial PINK1 and Mfn2 expression were reduced in the TAC model. In addition, our study showed that overexpression of PINK1 inhibited the expression of Mfn2. Therefore, PINK1 was involved in mitophagy induction in response to TAC-induced cardiac hypertrophy.

### 3.4. Mfn2 Overexpression Prevented Iso-Induced Cardiomyocyte Injury by Enhancing Mitochondrial Fusion

**Figure 4:**
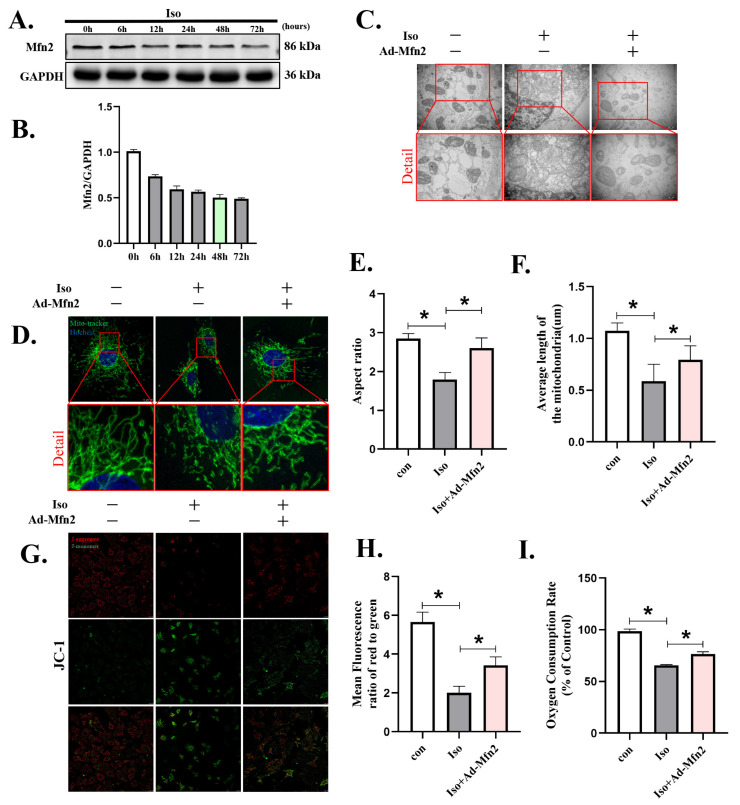
Overexpression of Mfn2 attenuated isoprenaline-induced cardiomyocyte injury by promoting mitochondrial fusion. Adenoviruses Ad-Mfn2 and Ad-control were used to transfect cardiomyocytes and then were stimulated with Iso (10 µM) for 48 h. (**A**,**B**) Western blot showing the expression of PINK1 over time. (**C**) Mitochondrial morphology in cardiomyocytes was shown by TEM. Details of mitochondrial structures are shown in the red frame. (**D**–**F**) Representative immunofluorescence images of mitochondrial morphology. Mitochondria are shown in green, and nuclei are shown in blue. (**G**,**H**) Fluorescence images of MMP was detected by JC-1 tracker. J-aggregate staining is shown in red and J-monomer staining is shown in green. (**I**) Mitochondrial respiration was measured using extracellular oxygen consumption assay kits to assess oxygen consumption rate. Statistical analyses (**A**–**I**) were performed by one-way ANOVAs followed by Dunnett’s test post-hoc tests. Data are presented as the mean ± SD. (n = 3). * *p* < 0.05.

Evidence has shown that Mfn2 promotes mitochondrial fusion and mitophagy and thus benefits the maintenance of mitochondrial network homeostasis. Here, we examined the effect of Mfn2 on Iso-treated cardiomyocytes. With increased Iso stimulation time, the expression of Mfn2 decreased gradually (Figure 4A,B, File S1). To detect the effects of Mfn2 overexpression on mitochondrial fusion, we examined mitochondrial changes by transmission electron microscopy (TEM) and confocal microscopy. TEM showed that Iso stimulation not only induced the swelling of the inner and outer mitochondrial membranes but also resulted in the loss of matrix material and vacuoles in the stroma (Figure 4C). However, this phenotype was alleviated after overexpression of Mfn2, and two small and short mitochondria underwent fusion to form large mitochondria. Fluorescence images of mitochondria visualized through microscopy revealed that after Iso administration, the mitochondria were changed from filamentous to rounded, and the aspect ratio and average length of the mitochondria were decreased (*p* < 0.05) (Figure 4D–F). After Mfn2 overexpression, mitochondrial elongation was induced, and the average length of the mitochondria was increased compared to the Iso group (*p* < 0.05). In addition, mitochondrial function was detected by JC-1 and oxygen consumption rate (OCR). JC-1 (G, H) staining showed significant descent of the MMP in Iso-treated cardiomyocytes. An OCR assay (Figure 4I) showed that mitochondrial respiratory function in cardiomyocytes treated with Iso was decreased compared to the control group. Overexpression of Mfn2 significantly restored the MMP (*p* < 0.01) induced by Iso and partially improved the OCR (*p* < 0.05). These results show that overexpression of Mfn2 ameliorated mitochondrial fragmentation by enhancing mitochondrial fusion.

### 3.5. Metformin Prevented Iso-Induced Cardiomyocyte Injury by Enhancing Mitochondrial Biogenesis

**Figure 5:**
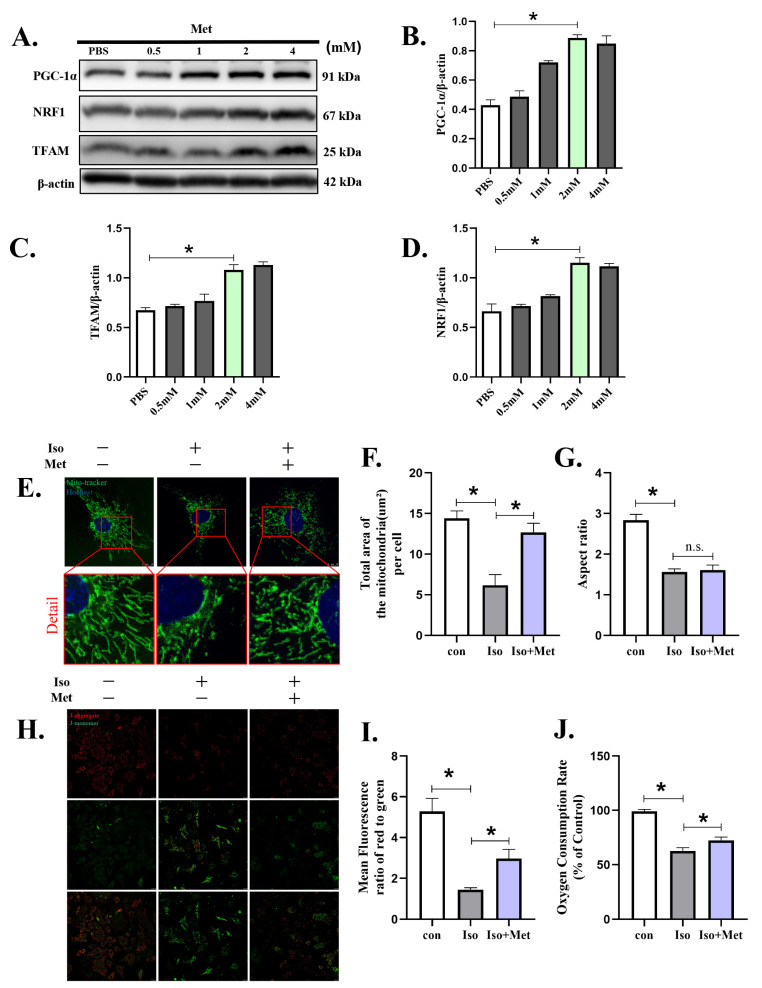
Metformin attenuated isoprenaline-induced cardiomyocyte injury by enhancing mitochondrial biogenesis. Metformin was used to transfect cardiomyocytes and then stimulated with Iso (10 µM) for 48 h. (**A**–**D**) Western blot showing the expression of PGC-1α, NRF1 and TFAM for the indicated time in cardiomyocytes. (**E**–**G**) Representative immunofluorescence images of mitochondrial morphology. Mitochondria are shown in green, and nuclei are shown in blue. (**H**,**I**) Fluorescence images of MMP was detected by JC-1 tracker. J-aggregate staining is shown in red and J-monomer staining is shown in green. (**J**) Mitochondrial respiration was measured using extracellular oxygen consumption assay kits by assessing oxygen consumption rate. Statistical analyses (**A**–**J**) were performed by one-way ANOVAs followed by Dunnett’s test post-hoc tests. Data are presented as the mean ± SD. (n = 3). * *p* < 0.05.

To verify the effects of metformin on mitochondrial biogenesis in cardiomyocytes, NRVMs were exposed to various concentrations of metformin (Figure 5A, File S1) and subjected to Western blotting. The expression of PGC-1α (Figure 5B), TFAM (Figure 5C) and NRF1 (Figure 5D), which represent the biogenesis of mitochondria, increased proportionally to the treatment concentration. To investigate the effects of metformin on mitochondria, we detected mitochondrial changes by confocal microscopy. Fluorescence images of mitochondria visualized through microscopy revealed that after Iso administration the total area of mitochondria decreased (Figure 5E–G). The mitochondria were changed from filamentous to rounded, which led to a reduction in the aspect ratio of the mitochondria (*p* < 0.05). As expected, metformin increased the biogenesis of mitochondria, as indicated by the total area (12.67 ± 1.13 vs. 6.18 ± 1.31 µm^2^, *p* < 0.01). However, the aspect ratio (1.61 ± 0.18 vs. 1.57 ± 0.07, *p* > 0.05) of the mitochondria was not significantly changed, and the mitochondria remained round. Additionally, mitochondrial function was detected by JC-1 and oxygen consumption rate (OCR). JC-1 (Figure 5H,I) staining shown severe impairment of the MMP in Iso-treated cardiomyocytes. An OCR assay (Figure 5J) showed that mitochondrial respiratory function in cardiomyocytes treated with Iso was significantly decreased compared to the control group. Metformin treatment significantly restored the MMP (*p* < 0.05) induced by Iso and partially improved the OCR (*p* < 0.05). These results showed that metformin ameliorated Iso-induced mitochondrial injury, which may partially occur through enhanced mitochondrial biogenesis by activating the PGC-1α pathway.

### 3.6. The Combination of PINK1, Mfn2 and Metformin Further Ameliorated NRVM Injury by Reducing ROS Generation and Apoptosis

Evidence has shown that mitochondrial quality control is achieved by well-coordinated mitochondrial biogenesis, mitochondrial dynamics and mitophagy, prompting us to explore a combined strategy in NRVMs. As shown in Figure S4A in the Supplementary Materials, compared to the Iso + Ad-control group (4.25 ± 0.5), the degree of mitochondrial–lysosomal overlap also increased in the Iso + Ad-PINK1 + Met group (7.5 ± 0.58, *p* < 0.05), which indicated that overexpressing PINK1 also increased mitophagy, while metformin increased mitochondrial biogenesis. In addition, Western immunoblotting to detect beclin1 (1.15 ± 0.04 vs. 0.92 ± 0.02, *p* < 0.05), p62 (0.27 ± 0.03 vs. 0.78 ± 0.04, *p* < 0.01) and LC3 (0.79 ± 0.05 vs. 0.61 ± 0.02, *p* < 0.05) supported the results (Figure S4B in the Supplementary Materials). In addition, compared to the Iso + Ad-PINK1 + Met group, overexpression of Mfn2 further increased mitochondrial lysosomal colocalization (9.75 ± 0.50 vs. 7.50 ± 0.58, *p* < 0.01) and LC3II expression (1.23 ± 0.06 vs. 0.79 ± 0.04, *p* < 0.01). The results showed that Mfn2 independently or collaboratively with PINK1 and metformin increased mitophagy. Fluorescence images showed that compared to the Iso + Ad-PINK1 group, in the Iso + Ad-PINK1 + Met group, although metformin increased the biogenesis of mitochondria, as indicated by the total area (11.9 ± 0.7 vs. 6.74 ± 0.47 µm^2^, *p* < 0.01), the aspect ratio (1.55 ± 0.12 vs. 1.58 ± 0.13, *p* > 0.05) and average length (0.46 ± 0.03 vs. 0.47 ± 0.05 µm, *p* > 0.05) of the mitochondria were not significantly changed, and the mitochondria remained round (Figure S4C in the Supplementary Materials). An increase in the average mitochondrial length (0.71 ± 0.09 vs. 0.46 ± 0.03 µm, *p* < 0.01) was observed after Mfn2 overexpression compared to the Iso +Ad-PINK1 + Met group. Notably, Western immunoblotting (Figure S4D in the Supplementary Materials) showed that, compared to the Iso + Ad-PINK1 + Met group, Mfn2 might exhibit a synergistic effect with metformin in mitochondrial biogenesis, as indicated by the expression of NRF-1 and PGC-1α.

**Figure 6:**
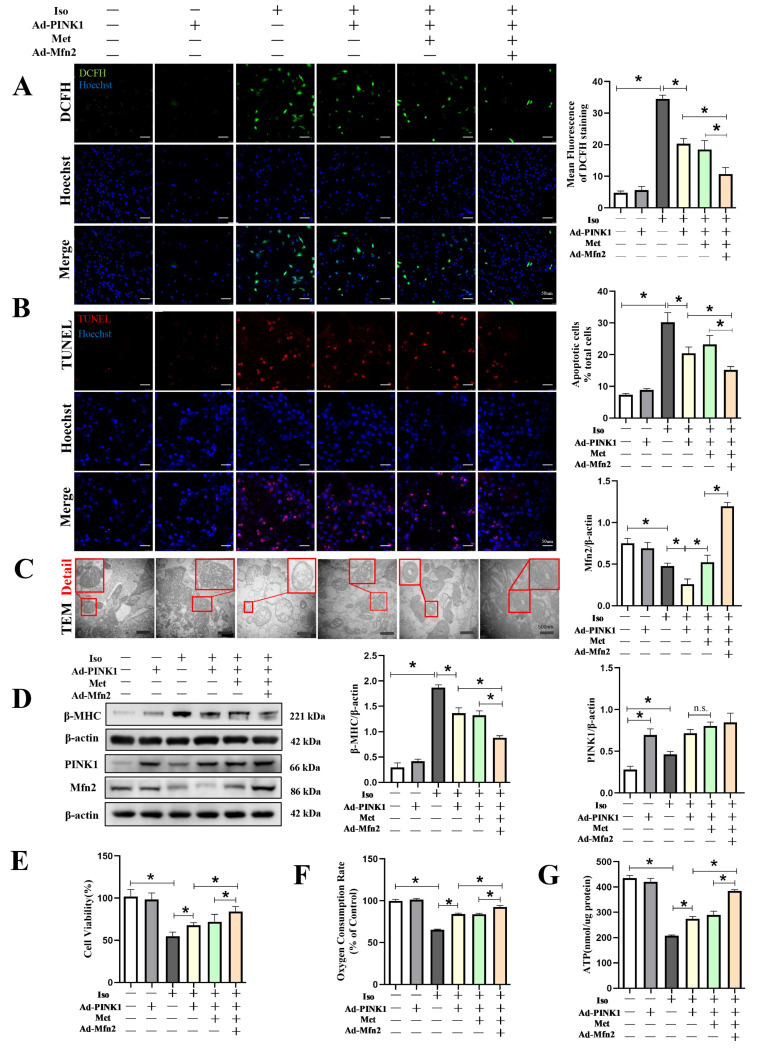
Mfn2 and Metformin in collaboration with PINK1 improved mitochondrial function and prevented Iso-induced cardiomyocyte injury. Cardiomyocytes were split into six groups: (1) control + Ad-control, cardiomyocytes were transfected with control adenovirus and were not treated with Iso; (2) control + Ad-PINK1, cardiomyocytes were treated with PINK1 adenovirus and were not treated with Iso; (3) Iso + Ad-control, cardiomyocytes were transfected with control adenovirus and treated with 10 µM Iso for 48 h; (4) Iso + Ad-PINK1, cardiomyocytes were transfected with PINK1 adenovirus and treated with 10 µM Iso for 48 h; (5) Iso + Ad-PINK1 + Met, cardiomyocytes were transfected with PINK1 adenovirus and treated with 10 µM Iso and 2 mM metformin for 48 h; and (6) Iso + Ad-PINK1 + Met + Ad-Mfn2, cardiomyocytes were transfected with PINK1 and Mfn2 adenovirus then treated with 10 µM Iso and 2 mM metformin for 48 h. (**A**) DCFH staining was used to show ROS production in cardiomyocytes. DCFH-positive cells are shown in green. (**B**) TUNEL staining was used to show apoptotic cardiomyocytes in each group. TUNEL-positive cells are shown in red. (**C**) Mitochondrial morphology is shown by TEM in cardiomyocytes. Details of mitochondrial structures are shown in the red frame. (**D**) Western blot showing the expression of β-MHC, PINK1 and Mfn2 in cardiomyocytes. (**E**) CCK-8 assay results show the cell viability in cardiomyocytes. (**F**) Mitochondrial respiration was measured using extracellular oxygen consumption assay kits by assessing oxygen consumption rate. (**G**) ATP assay kit with a luminometer was used to determine intracellular ATP levels. Statistical analyses (**A**–**G**) were performed by one-way ANOVAs followed by Bonferroni test post-hoc tests. Data are presented as the means ± SD, (n = 3). * *p* < 0.05.

The combination strategy further increased mitophagy and the expression of mitochondrial biogenesis-related proteins, as indicated in the aforementioned results. Therefore, we further tested the combination strategy with DCFH and TUNEL staining. Compared to the Iso + Ad-PINK1 + Met group, the combination strategy led to reduced ROS generation (10.7 ± 2.09 vs. 18.43 ± 2.90, *p* < 0.05, Figure 6A) and a lower apoptosis rate (15.2 ± 1.08% vs. 23.23 ± 2.80, *p* < 0.05, Figure 6B). In addition, representative TEM images of mitochondrial morphology, shown in Figure 6C, indicated that this strategy might not only attenuate Iso-induced swelling of the inner and outer mitochondrial membranes but might also ameliorate the loss of matrix material and vacuoles in the stroma. More importantly, this strategy profoundly inhibited the expression of beta-MHC (0.87 ± 0.11 vs. 1.33 ± 0.08, *p* < 0.05, Figure 6D, File S1), an essential protein that participates in cardiac hypertrophy. Use of adenovirus-mediated overexpression of PINK1 and Mfn2 protein induced corresponding overexpression of PINK1 and Mfn2. Compared to the Iso + Ad-PINK1 group, although the expression of PINK1 tended to increase after treatment with metformin, there was no statistical significance between the two groups. Immunoblotting showed that the expression levels of Mfn2 were decreased in the Iso + Ad-control group (*p* < 0.001), and overexpression of PINK1 could further attenuate the expression of Mfn2. Interestingly, the expression of Mfn2 was increased after cells were treated with metformin compared to the Iso + Ad-PINK1 group (Figure 6D). Therefore, these combined effects increased cell viability, as shown by CCK8 (84.22 ± 5.98 vs. 71.96 ± 8.89, *p* < 0.05, Figure 6E), oxygen consumption rate (92.94 ± 2.23 vs. 83.93 ± 0.87, *p* < 0.01, Figure 6F) and ATP generation (384.5 ± 4.93 nmol/µg vs. 289.75 ± 14.55, *p* < 0.01, Figure 6G) assays. These results suggested that the effects of the combination strategy are due to the amelioration of mitochondrial injury, which is probably caused by increased mitochondrial quality control and mitochondrial biogenesis.

## 4. Discussion

Activation of the sympathetic nervous system plays an important role in cardiovascular physiopathology [31,32,33]. As a vital neuroendocrine factor, Iso induces cardiac hypertrophy. Using an in vitro and in vivo hypertrophy model, we found that dysregulated mitochondrial quality control resulted in cardiomyocyte damage and death. We found that overexpression of PINK1 could increase mitophagy and alleviate Iso-induced cardiomyocyte injury and TAC-induced myocardial hypertrophy. We investigated a combination strategy of mitophagy and mitochondrial biogenesis induced by PINK1/Mfn2 dual overexpression and metformin stimulation. Compared to the Iso + Ad-PINK1 and Iso + Ad-PINK1 + Met groups, the combined strategy group showed not only the clearing of damaged mitochondria because of enhanced mitophagy but also increased mitochondrial biogenesis through the action of metformin, which replenished the lost healthy mitochondria. More importantly, the restoration of the structure and morphology of the mitochondrial network further enhanced mitochondrial function in Iso-treated NRVMs, indicating a new strategy for the treatment of cardiac hypertrophy.

Recent studies have indicated that mitochondrial dysfunction plays an important role in the development of myocardial hypertrophy. Hypertrophic cardiomyocytes exhibit abnormal mitochondrial structure and function, including damaged mitochondrial dynamics; reduced mitochondrial membrane potential, respiratory capacity, and ATP production; and increased ROS production. Furthermore, ROS activate the apoptotic signaling pathway, which eventually causes cardiomyocyte injury and, sometimes, death [5,34]. Therefore, it is crucial to attenuate mitochondrial dysfunction. A growing amount of evidence shows that PINK1/PARKIN/RBR E3 ubiquitin protein ligase-mediated mitophagy plays an important role in degrading damaged mitochondria in response to stress [35,36,37]. Hence, we overexpressed PINK1 in Iso-induced cardiomyocyte injury and observed that upregulation of PINK1 expression increased mitophagy, leading to the clearance of damaged mitochondria. Moreover, PINK1 overexpression reduced ROS levels and the apoptosis rate and enhanced mitochondrial respiratory function. More importantly, we constructed myocardial-specific overexpression in mice and found that cardiac-specific overexpression of PINK1 improved cardiac function, attenuated pressure overload-induced cardiac hypertrophy and fibrosis and facilitated myocardial mitophagy in TAC mice. On the basis of these findings, we confirmed that mitophagy plays a vital role during Iso-induced cardiac injury and TAC-induced cardiac hypertrophy by promoting the degradation of damaged mitochondria.

Although mitophagy removes damaged mitochondria and increases the efficiency of mitochondrial utilization, increasing the quantity of new mitochondria might also be beneficial, particularly because of the lack of energy in the heart caused by reduced mitochondrial ATP productivity and the dearth of healthy mitochondria [2,5]. In addition, previous studies indicated that mitochondrial removal and replenishment reached equilibrium allowing the maintenance of a set mitochondrial volume [38]. Thus, both mitochondrial elimination and mitochondrial biogenesis need to be emphasized, as they both play integral roles in satisfying the energy demands of the whole body.

PGC-1α is an important protein in promoting mitochondrial biogenesis, which is activated in response to the increase in energy demands resulting from fasting, exposure to cold or physical exercise [12,39]. Under these conditions, PGC-1α is a powerful transcriptional coactivator that increases the expression of various downstream genes that are involved in mitochondrial biogenesis. These transcription factors include nuclear respiratory factor (NRF1/2), peroxisome proliferator-activated receptor (PPAR) and estrogen-associated receptor (ERRS). NRF1 and NRF2 promote the expression of nuclear-encoded mitochondrial transcription factor A (TFAM), which is critical for mtDNA transcription [40]. Metformin acts as an AMPK activator that has been shown to increase the expression of PGC-1α, a strong transcriptional coactivator that promotes mitochondrial biogenesis [13,41]. Therefore, metformin was selected to promote mitochondrial biogenesis in our study. We observed that the protein expression of PGC-1α, NRF1 and TFAM was increased in a dose-dependent manner after metformin treatment. Additionally, compared to that in the Iso + Ad-PINK1 group, metformin activated mitochondrial regeneration-related proteins and increased the total area of mitochondria, which represented mitochondrial biogenesis. These results suggest that metformin can increase mitochondrial regeneration in both normal and Iso-damaged cells.

Although metformin can increase mitochondrial biogenesis, mitochondria were fragmented after metformin treatment, as indicated by the aspect ratio and average length. Moreover, no further elevation was observed in mitochondrial respiratory function or ATP production, possibly because the function of mitochondria is strongly associated with the dynamic mitochondrial network. Studies have shown that a proper dynamic balance between mitochondrial fusion and fission is fundamental to the function of mitochondrion [42]. Mitochondrial fragmentation often occurs when cardiomyocyte stress causes heart failure, and mitochondrial fission is intensified to separate the damaged mitochondria and facilitate their complete removal by mitophagy [43,44]. In addition, enhanced myocardial mitochondrial fission is closely related to the initiation of apoptosis [45]. Preventing mitochondrial fragmentation by reconstructing Mfn2 protects against cell death and heart failure [19]. Moreover, our research showed that Iso treatment reduced Mfn2 protein expression in a time-dependent manner. We found that Mfn2 overexpression could prevent Iso-induced cardiomyocyte injury by enhancing mitochondrial fusion. We upregulated the expression of Mfn2 with PINK1 overexpression and metformin stimulation. The results showed that this combination strategy exerted a beneficial effect on the mitochondrial network and cristae morphology, as reflected by the increased mitochondrial length and aspect ratio in Iso-treated cardiomyocytes relative to those in Iso + Ad-PINK1- and Iso + Ad-PINK1 + Met-treated cells. Restoration of mitochondrial homeostasis improved mitochondrial respiratory function and reduced ROS production. Thus, cell viability and cardiomyocyte apoptosis were further improved. Interestingly, we also found that overexpression of Mfn2 increased the expression of Beclin1, LC3 II, PGC-1α and NRF1 but decreased the expression of p62, which indicated that Mfn2 may cooperate with PINK1 to increase mitophagy and PGC-1α to increase mitochondrial regeneration.

In fact, previous studies have shown that the combination of mitophagy, mitochondrial regeneration and mitochondrial dynamics potentially ensures the health of the mitochondrial network [7,46]. However, mitochondrial function is severely damaged in cardiac hypertrophy or heart failure, and both mitochondrial biogenesis and mitophagy are inefficient. Therefore, both the elimination of damaged mitochondria and enhanced mitochondrial synthesis are required for improved cardiac function. Overall, these results indicate that the combination of PINK1, Mfn2 and metformin is crucial for overall mitochondrial quality control.

There were, however, some shortcomings in our study, including methodological problems. Previous studies have shown that metformin can reduce myocardial cell damage by increasing mitochondrial biogenesis. In our study, although the addition of metformin increased mitochondrial biogenesis on the basis of overexpression of PINK1, it did not further reduce cardiomyocyte injury. This is probably because the protective effect of metformin is masked by Ad-PINK1. More research is needed to explore the effects of simultaneous overexpression of PINIK1 and PGC-1α by transgenic technology. In addition, if we could achieve transgenic mice with the simultaneous overexpression of PINK1, PGC-1α and Mfn2, we would be able to verify our ideas in vivo. In addition, although we demonstrated the protective effects of Metformin in collaboration with PINK1/Mfn2 overexpression, its application in clinical practice needs further in-depth study.

## 5. Conclusions

In conclusion, the combined strategy of mitophagy and mitochondrial biogenesis by PINK1/Mfn2 and metformin improved overall mitochondrial quality and reduced the expression of cardiomyocyte damage markers. This strategy might be promising for the treatment of heart failure.

## Data Availability

The data presented in this study are available upon request from the corresponding author.

## Supplementary Materials

The following are available online at https://www.mdpi.com/article/10.3390/biology12040582/s1, Figure S1: The schematic diagram of mouse construction strategy and the typical graph of DNA running gel electrophoresis, Figure S2: Typical graphs showing blood flow velocity at the banding site of the aortic arch in sham and TAC groups, Figure S3: Overexpression of PINK1 attenuated cardiac hypertrophy in TAC mice, Figure S4: Combining PINK1, Mfn2 and metformin increased mitophagy and mitochondrial biogenesis; File S1: Original Images for Blots and Gels.
