# Supplementary material for: Metformin Collaborates with PINK1/Mfn2 Overexpression to Prevent Cardiac Injury by Improving Mitochondrial Function
Source: Biology (Basel). 2023 Apr 11;12(4):582. doi: 10.3390/biology12040582 (PMC10135998; doi:10.3390/biology12040582)
Supplement: Supplementary file 1 [file biology-12-00582-s001.zip › biology-2295924-Figure S1-S4.pdf]

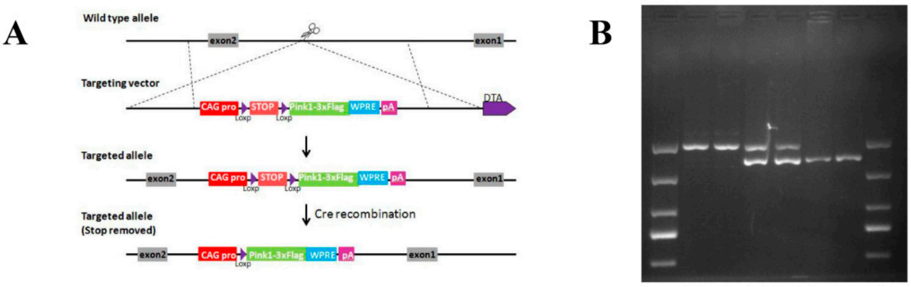

Figure S1. The schematic diagram of mouse construction strategy and the typical graph of DNA running gel electrophoresis. (A) The schematic diagram of mouse construction strategy. (B) The two on the left represent the wild type, the two in the middle represent heterozygotes, and the two on the right represent homozygotes.

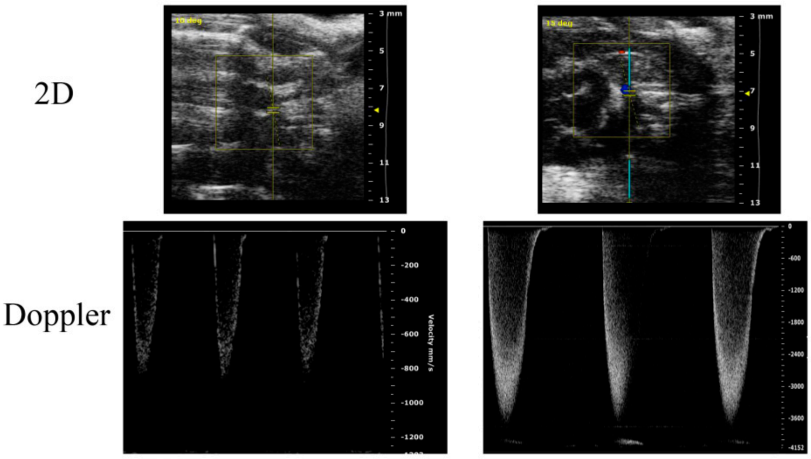

Figure S2. Typical graphs showing blood flow velocity at the banding site of the aortic arch in sham and TAC groups. Compared to the sham group, the blood flow velocity was significantly higher in TAC group.

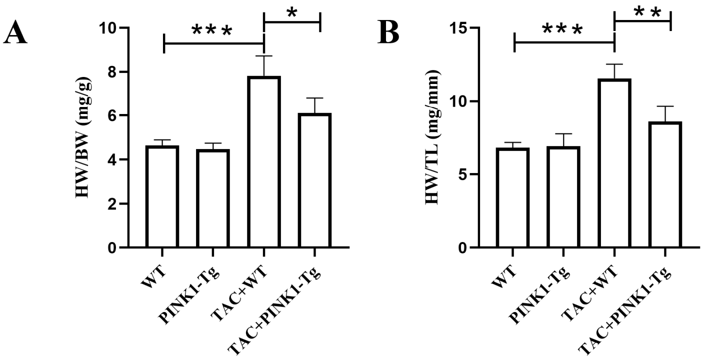

Figure S3. Overexpression of PINK1 attenuated cardiac hypertrophy in TAC mice. (A) Heart weight normalized to body weight(HW/BW). (B) Heart weight normalized to tibia length(HW/TL). Statistical analyses were performed by one-way ANOVAs followed by Bonferroni test post-hoc tests. *: $P < 0.05$, **: $P < 0.01$, ***: $P < 0.001$, values are presented as the mean ± SD, n = 6.

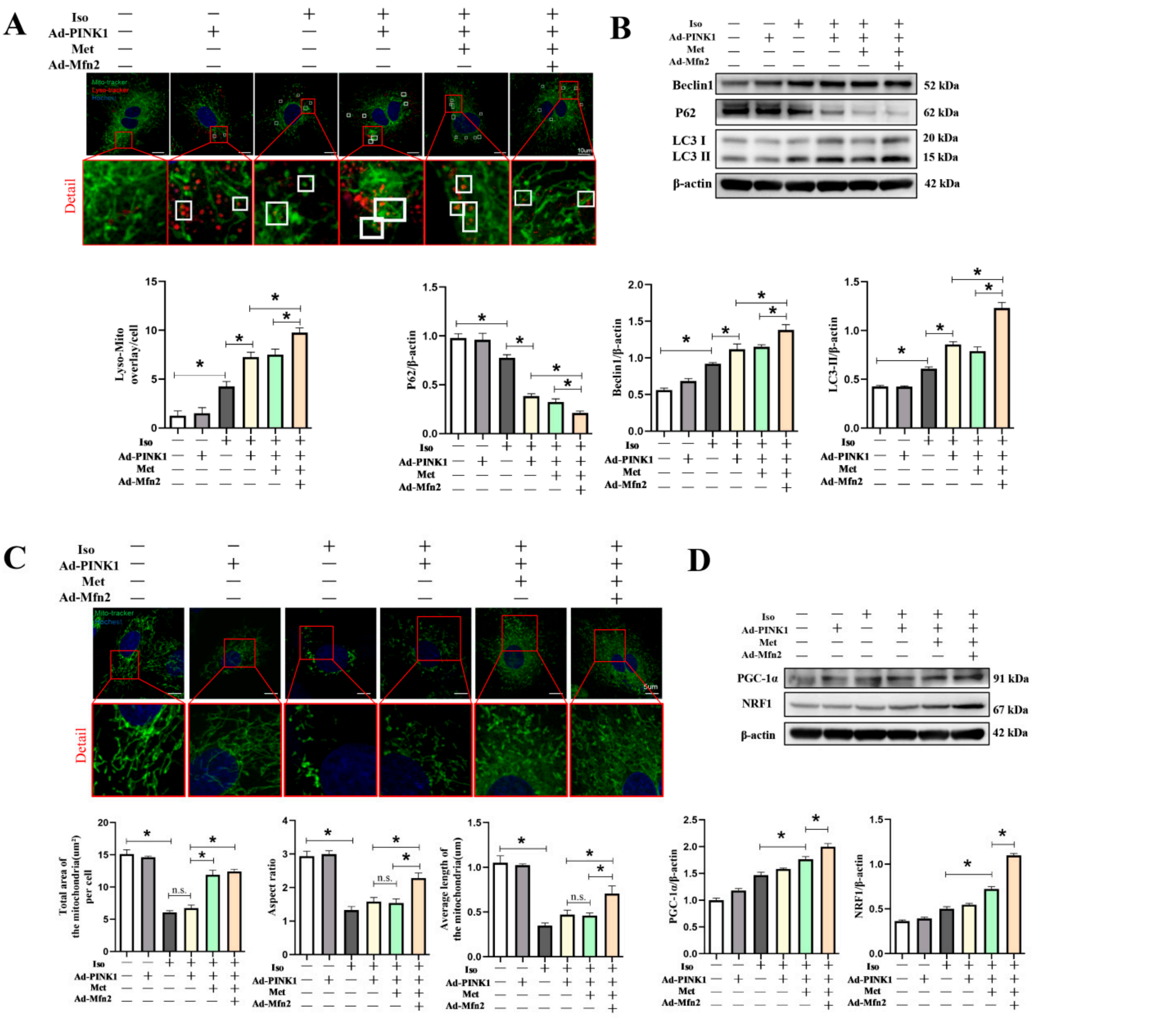

Figure S4. Combining PINK1, Mfn2 and metformin increased mitophagy and mitochondrial biogenesis. (**A**) Immunofluorescence images of lysosomal-mitochondrial interactions showing the effect of metformin and Mfn2 to mitophagy. An enlarged image shows the details of lysosomal-mitochondrial interactions in the red frame. (**B**) Immunoblotting showing the effect of metformin and Mfn2 to mitophagy in cardiomyocytes. (**C**) Mitochondrial morphology were detected by Mito-Tracker stain. Mitochondria are shown in green, nuclei were stained by Hoechst and shown in blue. An enlarged image shows the details of mitochondrial structures in the red frame. (**D**) Immunoblotting showing the effect of metformin on the expression of Mito-biogenesis related proteins in cardiomyocytes. Statistical analyses (**A**–**D**) were performed by one-way ANOVAs followed by Bonferroni test post-hoc tests. Data are presented as the mean ± SD. (n = 3). * $p < 0.05$.
