# Supplementary material for: Metformin Collaborates with PINK1/Mfn2 Overexpression to Prevent Cardiac Injury by Improving Mitochondrial Function
Source: Biology (Basel). 2023 Apr 11;12(4):582. doi: 10.3390/biology12040582 (PMC10135998; doi:10.3390/biology12040582)
Supplement: Supplementary file 1 [file biology-12-00582-s001.zip › biology-2295924-File S1 - Original Images for Blots and Gels.zip!/Explanantion.pdf]

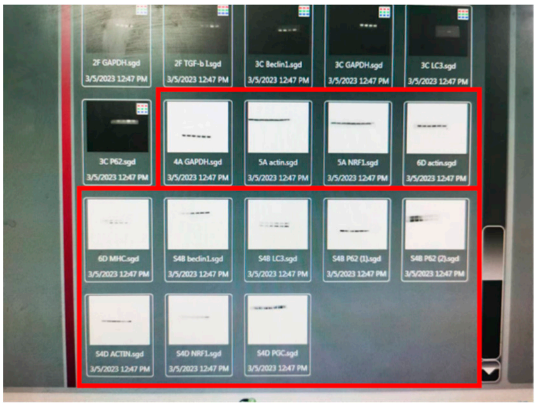

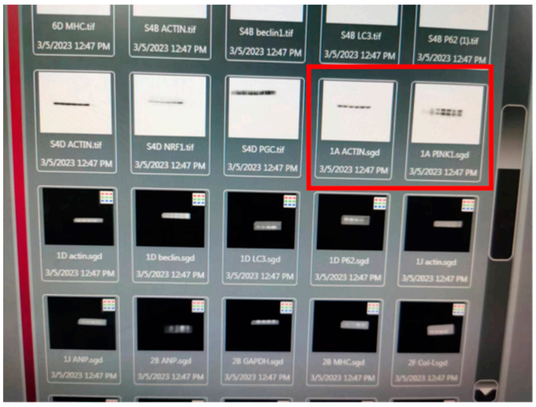

For some time, we found that there was a problem with the exposure machine in the laboratory. Although sgd format of pictures were saved in some western blot results, marker images were still not displayed. The picture in the red box is just that.
